# Brief Report: Lead Levels in Selected Electronic Cigarettes from Canada and the United States

**DOI:** 10.3390/ijerph15010154

**Published:** 2018-01-19

**Authors:** Zachary R. Dunbar, Ananth Das, Richard J. O’Connor, Maciej L. Goniewicz, Binnian Wei, Mark J. Travers

**Affiliations:** Roswell Park Comprehensive Cancer Center, Department of Health Behavior, Elm and Carlton Streets, Buffalo, NY 14263, USA; Zachary.Dunbar@roswellpark.org (Z.R.D.); ananthdas93@gmail.com (A.D.); richard.o’connor@roswellpark.org (R.J.O.); maciej.goniewicz@roswellpark.org (M.L.G.); Binnian.Wei@roswellpark.org (B.W.)

**Keywords:** e-cigarette, e-liquids, lead

## Abstract

Few published studies have investigated the presence of lead in the e-liquid of electronic nicotine delivery systems (ENDS). Lead inhalation is associated with increased risk of stroke, heart disease, and other diseases. This study used a novel application of graphite furnace technology to compare the concentration of lead between e-liquids of different packaging and product designs using e-liquids that are or were commercially available in the United States and Canada. Eleven nicotine-free disposable ENDS devices and 12 bottled refill solutions that contained nicotine were purchased from retailers in Canada and the United States between 2015 and 2017. E-liquids extracted from the disposable products and individual containers were analyzed for lead content by graphite furnace using atomic absorption detection. The lead concentration of open-wick ENDS devices ranged from 25.2 ppb to 838.4 ppb, with a standard deviation of 187.4 ppb. None of the bottled e-liquids contained quantifiable levels of lead. This study found that quantifiable levels of lead are present in certain disposable e-cigarette devices, and there is evidence from this study that the design of ENDS devices may contribute to lead exposure. These findings suggest that lead testing should be incorporated into future chemical analyses of ENDS devices.

## 1. Introduction

While the quantity of scientific literature on electronic nicotine delivery system (ENDS) constituents such as flavorings and carbonyls has grown immensely over the past five years, far less is known about levels of other potential toxicants, including toxic metals such as lead. In previous studies, significant amounts of lead have been quantified in a variety of different e-liquids, aerosol, and other components of ENDS devices [1,2,3,4]. Lead is an environmental pollutant that is associated with a multitude of deleterious health effects in exposed individuals. Lead exposure in the general population contributes to increased risk of heart disease, stroke, hypertension [5], renal damage [6], and issues with cognitive and/or behavioral development [7]. The action of lead as an antagonist of behavioral and cognitive growth is of particular concern to youths and adolescents, as approximately 1.7 million American high school students and 500,000 middle school students used e-cigarettes at least once in the past 30 days throughout 2016 [8]. Further, the Centers for Disease Control (CDC) has asserted that no safe blood level for lead has been established in children [9]. Despite the high volume of physiological and psychological reporting that has identified lead as a potent pollutant and public health risk, research on lead levels in e-cigarettes is still in its infancy, but it is crucial and urgent to perform an effective assessment of lead exposure in e-cigarette users, especially considering that the use of e-cigarettes is increasing in the United States and around the world.

Lead is a potential concern in ENDS for several reasons. Quantifiable levels of lead have been detected in components of conventional combustible tobacco [10]. *Nicotiana tabacum* (cultivated tobacco) is a potent bio-accumulator; therefore, pollutants such as heavy metals that are possibly present in the immediate growing environment may be absorbed by tobacco plants [11]. As the majority of pharmaceutical nicotine (which is used in e-liquid as well as other medicinal applications) originates from cultivated tobacco [12], lead may be introduced into the e-liquid of ENDS devices through the process of extracting nicotine from tobacco leaves. Similarly, there is the possibility that the materials from which components of the e-cigarette devices are constructed may introduce lead into the e-liquid of these devices [13]. Previous studies have also identified that the heating coil, wick, and other internal components of e-cigarette devices may contribute to the emission of lead in ENDS aerosol [14,15]. Therefore, the packaging and design of ENDS products may be an important contributor to lead exposure.

Disposable ENDS products with open wick designs are defined as products in which e-liquid is deposited onto a cotton or fabric pad that is tightly packaged inside the device. In open wick devices, the heating element of the device is in direct contact with the e-liquid at all times. Cartridge designs are defined as any ENDS device in which the e-liquid is encased in an interchangeable metal chamber, meaning that the e-liquid is in contact with the metal cartridge at all times, but never is directly exposed to the heating element of the device. Finally, e-liquid may also be purchased as refill solutions packaged in glass or plastic bottles; unless the e-liquid is removed from these containers, bottled e-liquids are never exposed to ENDS device elements. This study aims to employ graphite-furnace absorption spectroscopy to determine and compare the concentration of lead between e-liquids of different packaging and product designs using e-liquids that are or were commercially available in the United States and Canada.

## 2. Materials and Methods

The products selected for inclusion in this study were drawn as a convenience sample from a larger population purchased from Canadian or American tobacco retailers in the autumn of 2015, with the exception of JUUL brand products, which were purchased in spring of 2017. Products were selected for analysis according to the popularity of their flavorings, which was determined by simple frequency analysis using Wave 2 public-use data from the Population Assessment of Tobacco and Health (PATH) study [16]. The total number of products tested included 12 different e-liquids bottled in individual containers and 9 different e-liquids that were extracted from inside disposable ENDS products. Due to differences in labelling convention between products, expiration dates were only available for four bottled e-liquid products tested in this analysis: Blue V Watermelon (December 2015), Club Crazy Cola (July 2016), V Menthol (August 2015), and House of Vapor Menthol (February 2016). None of the disposable or cartridge e-cigarette systems were labelled with expiration dates. None of the bottled e-liquids had contact with ENDS devices prior to the analysis. All products were stored in a 4 °C refrigerator prior to chemical analysis. E-liquids from all disposable devices were labelled as nicotine-free, whereas e-liquids from the individual containers each contained nicotine, according to their respective product labels, as summarized below in Table 1.

E-liquid from disposable devices was extracted by isolation and transfer of the e-cigarette wick into 2 mL Eppendorf crimp-cap vials, followed by centrifugation (ThermoFisher Multifuge X1, Waltham, MA, USA) at 1000 rpm for approximately three seconds. 500 µL of e-liquid was then transferred from either the centrifuged Eppendorf vials or e-liquid bottles into labeled 1.2 mL total volume PTFE sample cups (PerkinElmer, Waltham, MA, USA). Next, 500 µL of 10% v/v aqueous nitric acid was transferred into each sample cup. The ultra-pure water used to create the diluted nitric acid solution was supplied by a MilliQ Integral Water Purification System (Millipore Sigma, Burlington, MA, USA). A calibration gradient for lead was generated using serial dilutions of a 1000 µg/mL lead standard (PerkinElmer, Waltham, MA, USA) and 10% v/v nitric acid (Millipore Sigma, Burlington, MA, USA). One aliquot was withdrawn per ENDS product, and this aliquot was assessed in triplicate. The geometric mean lead concentration was then calculated from the mean absorbance (*n* = 3) for each of the single e-liquid aliquots, and is reported below in units of in parts-per-billion (ppb).

All quantitative analysis was performed using a PerkinElmer 900Z graphite furnace atomic absorption spectrometer (GFAA) (PerkinElmer, Waltham, MA, USA). A 100 ppb lead calibration solution was generated by 10,000-fold dilution of a 1000 ppm ultra-purity lead standard solution (PerkinElmer, Waltham, MA, USA) using 10% v/v aqueous nitric acid. A calibration gradient was generated by 10, 5, 2.5, 1.6, and 1.25-fold dilutions of the 100 ppb calibration media, respectively, and each dilution was performed using the capillary system of the 900Z system using 10% v/v aqueous nitric acid as a diluent. Quality control assurance was performed by analysis of a 3.3, 2, and 1.4-fold dilutions of 100 ppb lead calibration matrix, and 20% variance from expected lead concentration was used as the threshold for successful quality control. Lead quantification was determined by calculation of a limit of quantitation (LOQ), which was defined as 10 times the standard deviation of the reagent blank divided by the slope of the calibration gradient [17]. SPSS Version 21 (IBM, Armonk, NY, USA) was used for chi-square, *t*-test, and ANOVA comparison assessments reported below. In the statistical analysis, an imputed value equal to $\frac{LOQ}{\sqrt{2}}$ represented samples with lead levels below the LOQ. The GFAA method used throughout this analysis is given below in Table 2 and Table 3.

## 3. Results

**Figure 1:**
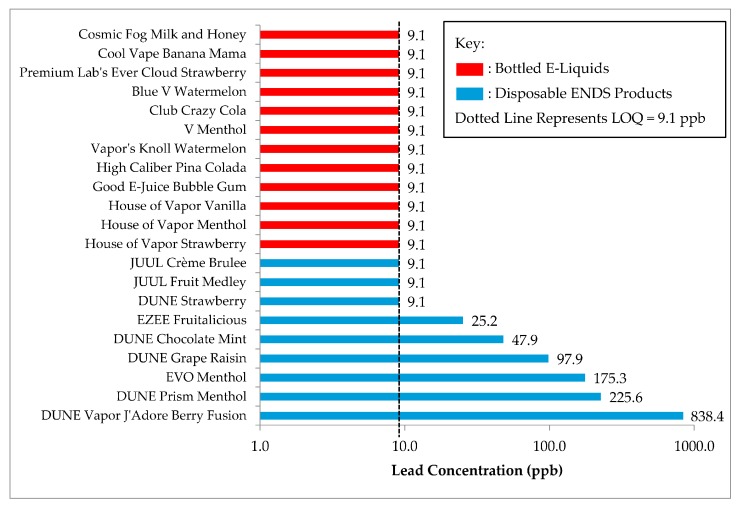
Lead concentration, by product.

The lead concentration of each ENDS product tested in this analysis is depicted below in Figure 1. Of the total product population, 5 disposable devices (41.9%) were of open-wick design, 4 disposable products (18.9%) were cartridge systems, and the remaining 12 products (39.2%) were bottled e-liquids. Approximately 30% (*n* = 5 open-wick, *n* = 1 cartridge system) of the products tested in this analysis contained quantifiable levels of lead. Open-wick ENDS devices were significantly more likely to contain detectable levels of lead compared to cartridge systems or bottled e-liquids (*χ*^2^: 17.3, *p* = 0.001). Disposable ENDS devices (geometric mean (GM) = 46.6 ppb, standard deviation (SD) = 5.9) contained significantly higher levels of lead on average compared to bottled e-liquids (*t* = 3.3, *p* = 0.010). Furthermore, there was a significant difference in lead concentration between open-wick devices (GM = 117.5 ppb, SD = 3.9) and cartridge (GM = 14.7 ppb, SD = 5.2) systems (*p* = 0.004). All of the products with lead levels above the limit of quantitation (9.1 ppb) originated from Canadian tobacco manufacturers, and none of the e-liquids that contained nicotine had quantifiable levels of lead.

## 4. Discussion

Among the samples collected in this study, none of the bottled e-liquids contained detectable levels of lead, which suggests that lead concentrations in disposable e-cigarettes may be related to the proximity of e-liquid to metal components in the product (e.g., solder). There was also a significant difference in lead concentration between cartridge and open-wick disposable systems, which suggests that the design of the ENDS products evaluated in this study contributed to overall lead exposure. Future research must be performed that investigates the mechanism through which lead enters e-liquid in e-cigarettes in order to better understand the public health risk posed by lead in ENDS devices.

All of the disposable products that contained detectable levels of lead were purchased at least one year prior to analysis. These findings suggest that lead levels may be elevated in certain ENDS devices, and the concentration of lead identified in each device may be related to the age of the product. Due to the absence of labeled expiration dates on the majority of products tested in this analysis, definitive claims about the interaction between product age and lead concentration cannot be made. However, this widespread absence of clearly labeled expiration dates demonstrates a need for more uniform labeling regulations on ENDS devices. Furthermore, although the U.S.A. Federal Food and Drug Administration (FDA) Deeming Rule [18] intends to closely regulate ENDS product labeling, there is no explicit language that mandates the inclusion of expiration dates on ENDS labels in either the FDA Deeming Rule or the FDA Guidance for Registration and Product Listing for Owners and Operators of Domestic Tobacco Product Establishments [19]. These findings highlight the need for better product labeling and future investigation of a possible time-dependent relationship between ENDS product age and the concentration of lead or other metals in these products; furthermore, the presence of other deleterious organic byproducts introduced into ENDS products through the oxidation of e-liquid over time should also be evaluated in future analyses.

The overall risk posed by lead in e-cigarettes may also depend upon product-usage characteristics such as the temperature of aerosol generation, other e-liquid ingredients, vaping puff topography, and total duration of use. As such, future studies of lead in ENDS aerosol, vaping behavior, and biological lead levels in users will help assess the risk posed by lead present in e-liquids. Further, existing exposure guidelines are imperfect comparison tools for assessing the risk posed by lead levels in ENDS devices. Although U.S.A. Environmental Protection Agency (EPA) standards exist for exposure to lead in drinking water [20] and ambient air [21], until more information is gathered regarding the expected dose of lead an average ENDS user may be exposed to, definitive claims about the toxicology and public health burden of lead in ENDS devices cannot be made. Overall, this study provides an important technique to investigate the concentration of lead in various e-liquids but does not allow for the definitive assessment of lead emitted in e-cigarette aerosol or cumulative risk associated with lead levels detected in these products.

Graphite furnace atomic absorption spectroscopy was an effective measurement device for the determination of lead concentration in e-liquid in this study. Although less than one-third of the samples tested above contained appreciable levels of lead, the quantitation limit of 9.11 ppb was sensitive enough to allow for differences between ENDS product designs to be investigated. GFAA technology is also relatively inexpensive and requires less intensive training to operate compared to more sophisticated instrumentation used in previous investigations into lead concentration in e-liquids and aerosol, such as inductively-coupled plasma mass-spectroscopy (ICP-MS) or scanning-electron microscopy (SEM), among others [1,2,3,4,14,15]. Furthermore, the reliance of this analysis on GFAA technology is in accordance with the proposed actions of the WHO Tobacco Laboratory Network (TobLabNet), which lists the “[improvement] of current laboratory capabilities to meet testing requirements [for tobacco products]” as a primary activity of the network [22]. Therefore, the successful implementation of GFAA for the assessment of lead levels in e-liquids is an important contribution to the wider field of heavy metal analysis in e-liquids, and is a major strength of this study. 

Other strengths of this study include the inclusion of a variety of different ENDS e-liquids and packaging types, and the assessment of products from both Canada and the United States. However, this study is subject to several important limitations. First, the total sample size of the analysis is limited. Second, although product age may be related to lead concentration in ENDS devices, this relationship cannot be accurately assessed using this study design, as lead content was only assessed at a single time point. Third, this analysis only investigated lead content in e-liquids, where other heavy metals such as chromium and nickel have also been quantified in ENDS devices [1]. Additionally, as only one physical product was assessed per product name, variance in lead content within a single product could not be investigated. Similarly, due to the preliminary nature of this analysis, definitive recommendations for product regulation and policy changes cannot be made from this data. However, despite these shortcomings, this analysis provides a novel method for the assessment of lead in e-liquids, and the preliminary data reported above raises important new questions for future research.

## 5. Conclusions

The identification of quantifiable levels of lead in the disposable e-cigarettes analyzed in this pilot study suggests that future investigations of potentially harmful compounds in e-cigarettes should include testing for lead and other heavy metals in order to ensure an adequate representation of possible public health risk associated with e-cigarette use. Furthermore, this pilot study suggests a need for future product standards to be developed that regulate the level of lead in ENDS device components. Future research should also investigate the physiological and public health impact of the presence of lead and other heavy metals such as chromium and nickel in e-cigarettes, and the mechanism by which heavy metals may be transferred from the e-liquid into the e-cigarette user must also be established. In all, this study contributes preliminary data that highlights important new avenues for future tobacco product research and regulation.

## Figures and Tables

**Table 1:** Products tested, by type.

**Disposable ENDS Devices**				
**Country of Origin**	**Brand**	**Product Name**	**Product Design**	**Label Nicotine Concentration (mg/mL)**
Canada	DUNE	Vapor J’Adore Berry Fusion	Open Wick	0
Canada	DUNE	Prism Menthol	Open Wick	0
Canada	DUNE	Grape Raisin	Open Wick	0
Canada	DUNE	Chocolate Mint	Open Wick	0
Canada	EZEE	Fruitalicious	Open Wick	0
Canada	EVO	Menthol	Open Wick	0
Canada	DUNE	Strawberry	Cartridge	0
United States	JUUL	Fruit Medley	Cartridge	0
United States	JUUL	Crème Brulee	Cartridge	0
**Bottled E-Liquids**				
**Country of Origin**	**Brand**	**Product Name**	**Package Design**	**Label Nicotine Concentration (mg/mL)**
United States	House of Vapor	Strawberry	Plastic	24
United States	House of Vapor	Menthol	Plastic	24
United States	House of Vapor	Vanilla	Plastic	24
United States	Good E-Juice	Bubblegum	Plastic	24
United States	High Caliber	Pina Colada	Plastic	24
United States	Vaper’s Knoll	Watermelon	Plastic	18
Canada	V	Menthol	Plastic	12
Canada	Club	Crazy Cola	Glass	18
Canada	Blue V	Watermelon	Plastic	12
Canada	Premium Labs’	Ever Cloud Strawberry	Plastic	12
Canada	Cool Vape	Banana Mama	Plastic	6
Canada	Cosmic Fog	Milk & Honey	Plastic	18

**Table 2:** Graphite furnace absorbance assessment conditions.

**Spectrometer Conditions**	
Element	Pb
λ (nm)	283.31
Slit Width (nm)	0.7
Signal	Background Corrected AA
Measurement	Peak Area
Lamp Type	Hollow Cathode
Gas Type	Argon (Ultra-Purity)
**Absorbance Read Parameters**	
Time (s)	5
Delay Time (s)	0
BOC Time (s)	2
Sample Volume (µL)	20

**Table 3:** Graphite furnace temperature program.

Step	Temperature (°C)	Ramp Time (s)	Hold Time (s)
1	110	1	30
2	130	15	30
3	850	10	20
4	1600	0	5
5	2450	1	3
